# Transcriptome sequencing revealed differences in the response of renal cancer cells to hypoxia and CoCl _2_ treatment

**DOI:** 10.12688/f1000research.7571.1

**Published:** 2015-12-30

**Authors:** Nadezhda Zhigalova, Artem Artemov, Alexander Mazur, Egor Prokhortchouk

**Affiliations:** 1 Institute of Bioengineering, Research Center of Biotechnology RAS, Moscow, Russian Federation

**Keywords:** Hypoxia, CoCl2, renal cancer, gene expression, metabolism pathways

## Abstract

Human cancer cells are subjected to hypoxic conditions in many tumours. Hypoxia causes alterations in the glycolytic pathway activation through stabilization of hypoxia-inducible factor 1. Currently, two approaches are commonly used to model hypoxia: an alternative to generating low-oxygen conditions in an incubator, cells can be treated with CoCl _2_. We performed RNA-seq experiments to study transcriptomes of human Caki-1 cells under real hypoxia and after CoCl _2_ treatment. Despite causing transcriptional changes of a much higher order of magnitude for the genes in the hypoxia regulation pathway, CoCl _2_ treatment fails to induce alterations in the glycolysis / gluconeogenesis pathway. Moreover, CoCl _2_ caused aberrant activation of other oxidoreductases in glycine, serine and threonine metabolism pathways.

## Introduction

Hypoxia is characterized by reduced oxygen supply and appears in multiple pathological conditions including tumours. However, hypoxia can also have a functional role during normal mammalian development and embryogenesis ^1^. Cells respond to hypoxic conditions both on biochemical and gene expression levels by switching from aerobic metabolism to anaerobic glycolysis and by expression of stress-related genes involved in regulation of cell death, erythropoiesis, angiogenesis and survival ^2– 4^. The activation of many O _2_-regulated genes is mediated by hypoxia-inducible factor (Hif1a). Under normoxia, Hif1a is hydroxylated by specific prolyl hydroxylases (PHD1, PHD2 and PHD3). This reaction requires oxygen, 2-oxoglutarate and ascorbate ^5, 6^. When Hif1a is hydroxylated, it interacts with the von Hippel-Lindau tumor suppressor protein (pVHL). pVHL forms the substrate-recognition module of an E3 ubiquitin ligase complex, which directs Hif1a poly-ubiquitylation and proteasomal degradation ^7, 8^. Under hypoxia (less than 5% O _2_), PHD activity is inhibited by cytoplasmic reactive-oxygen species (ROS) which alter the oxidation state of Fe ^2+^ (a cofactor for PHD activity) to Fe ^3+^. This alteration inhibits PHD activity and Hif1a hydroxylation, thus Hif1a cannot interact with pVHL and promotes HIf1a stabilization ^9, 10^. This anaerobic condition and stabilization of Hif1a are characteristic of many tumors. The most common molecular abnormality in renal cell carcinoma is the loss of VHL, which is found in about 50–70% of sporadic cases. Consequently, renal carcinomas with mutations in VHL have high steady-state levels of Hif1a expression and are hypoxic ^11^. Some divalent cations such as cobalt (Co ^2+^), nickel (Ni ^2+^), and the iron-chelator deferoxamine (DFX), have been applied to mimic hypoxic conditions in cultured cells as they activate hypoxic signals by stabilizing HIF1a ^12^. Transition metal Co ^2+^ could induce hypoxic response by inhibiting PHD activity via iron replacement. Therefore, treatment of a cell culture with cobalt chloride (CoCl _2_) is a common model of hypoxia ^13^. The second classical setup to study hypoxia is hypoxia induction in a CO _2_ incubator with a regulated level of oxygen (less than 1% O _2_). In this work, we performed RNA sequencing of Caki-1 clear cell renal cancer cell lines treated with hypoxia and with CoCl _2_ to understand how adequate CoCl _2_ treatment was as a hypoxia model. We propose that CoCl _2_ is not a completely correct model for hypoxia, as it aberrantly induces various hydroxylases not involved in hypoxia pathways and fails to induce downstream biochemical pathways normally induced by hypoxia.

## Methods

### Cell culture

Caki-1 human clear cell renal carcinoma cells were obtained from American Type Culture Collection (ATCC). Caki-1 cells were cultured in Dulbecco’s modified Eagle’s medium (DMEM) supplemented with 10% FBS (GIBCO). For hypoxia treatment, we placed cells into a CO _2_ incubator with O _2_ control (BINDER CO _2_ CB 53) with a regulated environment of 1% O _2_, 5% CO _2_ and 94% N _2_, or cobalt chloride (CoCl _2_, Sigma) 300 mkM (stock solution 100mM in water) for 24 h.

### RNA preparation and RNA sequencing

Total RNA was extracted from Caki-1 cells with Trisol reagent according to the manufacturer’s instructions (Invitrogen). Quality was checked with BioAnalyser and RNA 6000 Nano Kit (Agilent). PolyA RNA was purified with Dynabeads ^®^ mRNA Purification Kit (Ambion). An Illumina library was made from polyA RNA with NEBNext ^®^ mRNA Library Prep Reagent Set (NEB) according to the manual. Sequencing was performed on HiSeq1500 with 50 bp read length. 10 million reads were generated for each sample.

### Data analysis

Reads were mapped to hg19 genome (bowtie2-indexed reference downloaded from ftp://ftp.ccb.jhu.edu/pub/data/bowtie2_indexes/hg19.zip) with tophat2 software (version 2.1.0) ^14^. Gene models of non-overlapping exonic fragments ( http://www-huber.embl.de/pub/DEXSeq/analysis/encode/hsa.DEXSeq.gtf) were taken from ENSEMBL 54 database ( http://www.ensembl.org/). For each exonic fragment, total coverage by mapped reads in each sample was calculated with bedtools multicov tool (version 2.17.0). Total gene coverage was calculated as a sum of coverages of all non-overlapping exonic fragments of a gene. Differential expression analysis was performed by applying default read count normalization (estimateSizeFactors) and performing per-gene negative binomial tests (nbinomTest), implemented in DESeq R package (version 1.22.0), with default parameters ^15^.

We considered a gene to be differentially expressed if the adjusted p-value in DESeq test was lower than 0.05 and fold-change values were higher than 2 (or lower than $\frac{1}{2}$). These sets of differentially expressed genes were further used for gene category enrichment analysis. We took the subset of genes which were found differential in both hypoxia against normoxia controls and after CoCl _2_ treatment against normal control and only in one of each experiments. These 3 sets of genes were analyzed with DAVID web service (version 6.7) ^16^ to find KEGG ^17^ pathways enriched with the genes.

TCGA data on transcriptomes of kidney tumours (KIRC cohort) was downloaded from Broad Institute FireBrowse ( http://gdac.broadinstitute.org/runs/stddata__2015_11_01/data/KIRC/20151101/gdac.broadinstitute.org_KIRC.Merge_rnaseqv2__illuminahiseq_rnaseqv2__unc_edu__Level_3__RSEM_genes_normalized__data.Level_3.2015110100.0.0.tar.gz). Principal component analysis (PCA) was performed with R prcomp function.

A simple transcriptome-based hypoxia signature was constructed as follows: for every sample being evaluated (e.g., TCGA cancer sample), we considered only the genes which were differentially expressed between hypoxia and untreated Caki-1 cell line (DESeq test adjusted p-value< 0.05). For these genes, we multiplied their logarithmic fold-change (hypoxia vs untreated) to their expression in the evaluated sample. The resulting values were then summed up over the genes under consideration. This yielded a per-sample hypoxia score which would be higher in samples with increased expression of hypoxia-induced genes and decreased expression of hypoxia-suppressed genes.

## Results

Raw per-gene expression counts for individual genes (see Methods) The samples are labelled as untreated_1, untreated_2, untreated_3, CoCl _2__1, CoCl _2__2, CoCl _2__3, hypoxia_1, hypoxia_2, hypoxia_3 which correspond to 3 untreated samples, 3 samples treated with CoCl _2_ and 3 samples in hypoxic conditions. Click here for additional data file. Copyright: © 2015 Zhigalova N et al. 2015 Data associated with the article are available under the terms of the Creative Commons Zero "No rights reserved" data waiver (CC0 1.0 Public domain dedication).

To compare transcriptional effects caused by hypoxia and by CoCl _2_ exposure, we performed transcriptome sequencing (RNA-seq) of Caki-1 clear cell renal carcinoma cell line in three conditions: untreated, treated with CoCl _2_, and exposed to hypoxic conditions (1% O _2_). We searched for differentially expressed genes in two comparisons: CoCl _2_-treated against untreated cells and cells under hypoxia against untreated cells.

**Figure 1.**
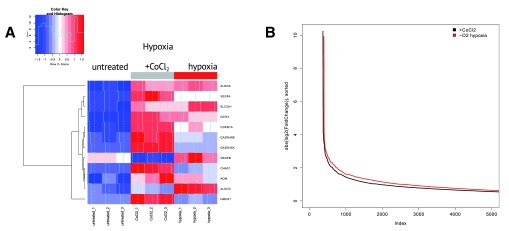
( **A**) CoCl _2_ causes much more pronounced expression changes in the expression of key hypoxia regulators compared to real hypoxia treatment. Heatmap represents relative gene expression for key genes involved in hypoxia regulation. ( **B**) Hypoxia results in broader transcriptome response compared to CoCl _2_ treatment, i.e., more genes are changing expression under hypoxia. The figure shows absolute log fold change values for gene expression between hypoxia (or CoCl _2_) groups relative to control group. Genes are sorted according to absolute log fold change values. *P*( *wilcoxon*) < 2.2 × 10 ^-16^.

We first asked how transcriptomic changes after both treatments fit into the established hypoxia gene signature ^1, 18, 19^. Figure 1A shows relative expression of the genes from hypoxia signature in all sequenced samples. We observed that genes in hypoxia signature were upregulated both in hypoxia and after CoCl _2_ treatment, though the effect was much stronger in CoCl _2_-treated samples. To check if a higher magnitude of transcriptomic changes in CoCl _2_ compared to hypoxic conditions was characteristic to other differentially expressed genes, we compared the distributions of logarithmic fold-changes (absolute value) for gene expression in these two experiments ( Figure 1B). Surprisingly, for the majority of genes, hypoxia-induced changes were higher than the ones induced by CoCl _2_. In other words, hypoxia resulted in broader transcriptome response than CoCl _2_ treatment even though specific hypoxia-related genes were more affected after CoCl _2_ treatment.

**Figure 2.**
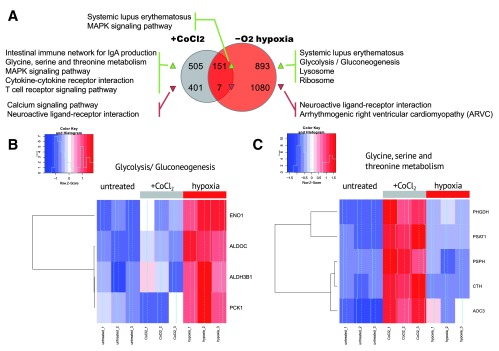
( **A**) Summary of KEGG pathways, enriched by the genes, up- and down-regulated in CoCl _2_ and hypoxia treatment. No enriched pathways were discovered for the genes downregulated in both treatments. ( **B**) Overall expression change in glycolysis/gluconeogenesis (KEGG hsa00260) genes in control, hypoxia conditions and after CoCl _2_ treatment. Glycolysis/Gluconeogenesis (KEGG hsa00010) is activated in hypoxia but not after CoCl _2_ treatment. ( **C**) Expression change for glycine, serine and threonine metabolism genes (KEGG hsa00010). Glycine, serine and threonine metabolism (KEGG hsa00260) is activated only after CoCl _2_ treatment but not under hypoxia.

These results suggested that CoCl _2_ treatment could be an incomplete model of hypoxia capturing only upstream signalling events in hypoxia pathways and not reflecting broader downstream effects. To further investigate the differences between CoCl _2_ model and real hypoxia, we compared the sets of differentially up- and down-regulated genes in CoCl _2_-treatment (against untreated) and in hypoxia-treated (against untreated) cells. Figure 2A summarizes the overlap between those gene sets. To understand what regulatory and biochemical pathways were affected in each treatment, we performed gene category enrichment analysis over KEGG ^17^ pathways with DAVID web service (version 6.7) ^16^ for genes affected in both treatments or exclusively in one treatment.

The genes which were significantly upregulated in hypoxic conditions (1% O _2_) but not after treatment with CoCl _2_ were significantly enriched in the glycolysis/gluconeogenesis pathway which was known to be related to hypoxia ^1, 15, 18^. Unexpectedly, we detected no enrichment in glycolysis/gluconeogenesis pathway with the genes differentially expressed after CoCl _2_ treatment which was confirmed by a heatmap for glycolysis/gluconeogenesis-related genes ( Figure 2B).

The genes upregulated in both hypoxia and under CoCl _2_ treatment were enriched in the MAPK pathway. As the MAPK pathway was known to activate hypoxic response, MAPK activation was expected in hypoxia. Even though CoCl _2_ affected directly HIF1a pathway, MAPK was activated in CoCl _2_-treated samples as well as in real hypoxia. This result supported a previous observation of MAPK-dependent activation of hypoxia response under CoCl _2_ treatment ^12^. Surprisingly, we observed systemic lupus erythematosus-related pathway activation in both treatments. The set of genes upregulated in this pathway (histone proteins H2A, H2B, H3, H4 and MHCII antigen-presenting genes) could be unrelated to lupus erythematosus, but rather could indicate increased proliferation and inflammation.

We also explored the genes specific to CoCl _2_ treatment but not affected by hypoxia. Surprisingly, the genes upregulated after CoCl _2_ treatment but not changed in hypoxia were enriched in the glycine/serine/threonine biosynthesis pathway ( Figure 2A and 2B). We hypothesized that Co ^2+^ ion could substitute metal cofactors of several oxidoreductases in the pathway and subsequently impair their activity. This, in turn, could require greater amounts of enzyme to be synthesized.

**Figure 3.**
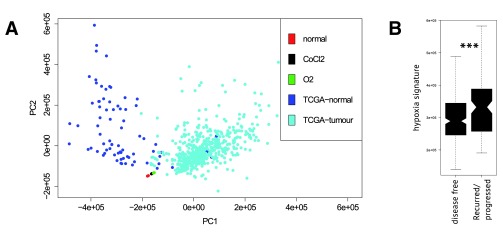
( **A**) PCA plot for TCGA samples of kidney tumours, adjacent normal tissue samples, untreated Caki-1 cells, Caki-1 cells treated with CoCl _2_ and the cells in hypoxic environment. ( **B**) Hypoxia signature derived from RNA-seq results predicted significantly higher hypoxia scores for recurred or progressed TCGA tumours.

Hypoxic conditions within a tumour have been shown to predict worse clinical outcome ^20^. We used our data on whole-transcriptome profiling of kidney cancer cell lines in normal and hypoxic conditions to extract a wide hypoxia signature and validate it with TCGA data on transcriptomes of kidney tumours ^21^. Major variation in TCGA transcriptomes ( Figure 3A) is generated by the difference between tumours and adjacent normal samples. We projected our sequenced samples to the principal components derived from TCGA samples and observed that the direction of transcriptome changes between untreated and hypoxic cell lines is slightly similar to the difference between normal and tumour samples, though hypoxia-related changes couldn’t explain normal-tumour difference. We constructed a transcriptome-based hypoxia signature as described in the Methods section. To test if this hypoxia signature predicted clinical outcome for kidney cancer patients, we explored the distribution of our hypoxia scores between disease free patients and patients in which the disease had recurred or progressed. Hypoxia scores were significantly higher for recurred/progressed patients (Wilcoxon test p-value=0.0009, Figure 3B).

## Discussion

The current study for the first time provides RNA-seq data revealing hypoxia-induced transcriptomic changes, which allows broader understanding of the processes related to hypoxia. In our analysis, we explored the limits of applicability of CoCl _2_ treatment as the model of hypoxia. Briefly, we observed that CoCl _2_ strongly alters expression of few genes important for hypoxia signalling, but fails to influence the essential downstream consequences of hypoxia, particularly the glycolysis/gluconeogenesis pathway. This might suggest the existence of alternative regulation mechanisms which trigger the downstream events in hypoxia together with main VHL/HIF1a pathway. CoCl _2_ treatment also abberantly induced pathways which did not respond to hypoxia. These included the glycine, serine and threonine metabolism pathways. We hypothesized that its aberrant activation might be caused by Co ^2+^ ion binding to the enzymes (other than PHD proteins) involved in Glycine, Serine and Threonine biosynthesis.

## Data availability

The data referenced by this article are under copyright with the following copyright statement: Copyright: © 2015 Zhigalova N et al.

Data associated with the article are available under the terms of the Creative Commons Zero "No rights reserved" data waiver (CC0 1.0 Public domain dedication). 

*F1000Research*: Dataset 1. Raw per-gene expression counts for individual genes (see Methods), 10.5256/f1000research.7571.d109570 ^23^

RNA-seq data was deposited to NCBI SRA under SRP066934 study accession code. The study contained experiments under the following accession codes: untreated (SRX1459966, SRX1459967, SRX1459969), treated with CoCl _2_ (SRX1459974, SRX1459977, SRX1459978), exposed to hypoxia (SRX1459979, SRX1459981, SRX1459984).

## References

[1] Hong SS Lee H Kim KW: HIF-1alpha: a valid therapeutic target for tumor therapy. *Cancer Res Treat.* 2004;36(6):343–353. 10.4143/crt.2004.36.6.343 20368827 PMC2843877

[2] Höckel M Vaupel P: Tumor hypoxia:definitions and current clinical, biologic, and molecular aspects. *J Natl Cancer Inst.* 2001;93(4):266–76. 10.1093/jnci/93.4.266 11181773

[3] Pouysségur J Dayan F Mazure NM: Hypoxia signalling in cancer and approaches to enforce tumour regression. *Nature.* 2006;441(7092):437–443. 10.1038/nature04871 16724055

[4] Yee Koh M Spivak-Kroizman TR Powis G: HIF-1 regulation: not so easy come, easy go. *Trends Biochem Sci.* 2008;33(11):526–534. 10.1016/j.tibs.2008.08.002 18809331

[5] Hu CJ Wang LY Chodosh LA: Differential roles of hypoxia-inducible factor 1alpha (HIF-1alpha) and HIF-2alpha in hypoxic gene regulation. *Mol Cell Biol.* 2003;23(24):9361–9374. 10.1128/MCB.23.24.9361-9374.2003 14645546 PMC309606

[6] Ivan M Kondo K Yang H: HIFalpha targeted for VHL-mediated destruction by proline hydroxylation: implications for O _2_ sensing. *Science.* 2001;292(5516):464–8. 10.1126/science.1059817 11292862

[7] Maxwell PH Wiesener MS Chang GW: The tumour suppressor protein VHL targets hypoxia-inducible factors for oxygen-dependent proteolysis. *Nature.* 1999;399(6733):271–5. 10.1038/20459 10353251

[8] Tanimoto K Makino Y Pereira T: Mechanism of regulation of the hypoxia-inducible factor-1 alpha by the von Hippel-Lindau tumor suppressor protein. *EMBO J.* 2000;19(16):4298–309. 10.1093/emboj/19.16.4298 10944113 PMC302039

[9] Hagen T Taylor CT Lam F: Redistribution of intracellular oxygen in hypoxia by nitric oxide: effect on HIF1alpha. *Science.* 2003;302(5652):1975–1978. 10.1126/science.1088805 14671307

[10] Simon MC: Mitochondrial reactive oxygen species are required for hypoxic HIF alpha stabilization. *Adv Exp Med Biol.* 2006;588:165–170. 10.1007/978-0-387-34817-9_15 17089888

[11] Thomas GV Tran C Mellinghoff IK: Hypoxia-inducible factor determines sensitivity to inhibitors of mTOR in kidney cancer. *Nat Med.* 2006;12(1):122–7. 10.1038/nm1337 16341243

[12] Triantafyllou A Liakos P Tsakalof A: Cobalt induces hypoxia-inducible factor-1alpha (HIF-1alpha) in HeLa cells by an iron-independent, but ROS-, PI-3K- and MAPK-dependent mechanism. *Free Radic Res.* 2006;40(8):847–56. 10.1080/10715760600730810 17015263

[13] Piret JP Mottet D Raes M: CoCl _2_, a chemical inducer of hypoxia-inducible factor-1, and hypoxia reduce apoptotic cell death in hepatoma cell line HepG2. *Ann N Y Acad Sci.* 2002;973:443–447. 10.1111/j.1749-6632.2002.tb04680.x 12485908

[14] Kim D Pertea G Trapnell C: TopHat2: accurate alignment of transcriptomes in the presence of insertions, deletions and gene fusions. *Genome Biol.* 2013;14(4):R36. 10.1186/gb-2013-14-4-r36 23618408 PMC4053844

[15] Anders S Huber W: Differential expression analysis for sequence count data. *Genome Biol.* 2010;11(10):R106. 10.1186/gb-2010-11-10-r106 20979621 PMC3218662

[16] Huang da W Sherman BT Lempicki RA: Systematic and integrative analysis of large gene lists using DAVID bioinformatics resources. *Nat Protoc.* 2009;4(1):44–57. 10.1038/nprot.2008.211 19131956

[17] Kanehisa M Goto S: KEGG: kyoto encyclopedia of genes and genomes. *Nucleic Acids Res.* 2000;28(1):27–30. 10.1093/nar/28.1.27 10592173 PMC102409

[18] Benita Y Kikuchi H Smith AD: An integrative genomics approach identifies Hypoxia Inducible Factor-1 (HIF-1)-target genes that form the core response to hypoxia. *Nucleic Acids Res.* 2009;37(14):4587–602. 10.1093/nar/gkp425 19491311 PMC2724271

[19] Eustace A Mani N Span PN: A 26-gene hypoxia signature predicts benefit from hypoxia-modifying therapy in laryngeal cancer but not bladder cancer. *Clin Cancer Res.* 2013;19(17):4879–88. 10.1158/1078-0432.CCR-13-0542 23820108 PMC3797516

[20] Jubb AM Buffa FM Harris AL: Assessment of tumour hypoxia for prediction of response to therapy and cancer prognosis. *J Cell Mol Med.* 2010;14(1–2):18–29. 10.1111/j.1582-4934.2009.00944.x 19840191 PMC3837600

[21] The Cancer Genome Atlas Research Network: Comprehensive molecular characterization of clear cell renal cell carcinoma. *Nature.* 2013;499(7456):43–49. 10.1038/nature12222 23792563 PMC3771322

[22] Weigand JE Boeckel JN Gellert P: Hypoxia-Induced alternative splicing in endothelial cells. *PLoS One.* 2012;7(8):e42697. 10.1371/journal.pone.0042697 22876330 PMC3411717

[23] Zhigalova N Artemov A Mazur A: Dataset 1 in: Transcriptome sequencing revealed differences in the response of renal cancer cells to hypoxia and CoCl _2_ treatment. *F1000Research.* 2015 Data Source 10.12688/f1000research.7571.1 PMC4712771 26925226

