# Supplementary material for: Transcriptome sequencing revealed differences in the response of renal cancer cells to hypoxia and CoCl 2 treatment
Source: F1000Res. 2015 Dec 30;4:1518. [Version 1] doi: 10.12688/f1000research.7571.1 (PMC4712771; doi:10.12688/f1000research.7571.1)
Supplement: Peer review history

# Peer review: Referee response for version 1

**DOI:** 10.5256/f1000research.8152.r11907

**Published:** 2016-01-12

**Authors:** Colyn Crane-Robinson

**Affiliations:** 1 School of Biological Sciences, Biophysics Laboratories, University of Portsmouth, Portsmouth, UK

This project uses mRNA sequencing to monitor changes in gene expression in a human kidney cell line in response to direct hypoxia (5% O _2_) as compared to treatment with 300mM CoCl _2_, frequently used as a surrogate for oxygen depletion. The results are very clear-cut: monitoring several genes known to be key hypoxia regulators shows CoCl _2_ to be more effective than oxygen depletion but in contrast, monitoring a much broader gene response showed O _2_ depletion to have greater effects. This contrast led the authors to concentrate attention on several specific biochemical pathways: a) glycolysis/gluconeogenesis is strongly activated in hypoxia (as expected) but not following treatment with CoCl _2_; b) the reverse is the case for glycine, serine, threonine metabolism which is strongly induced by CoCl _2 _but not by hypoxia.

These last examples make clear the non-equivalence of CoCl _2 _treatment and real hypoxia and this article therefore issues a strong caveat regarding the use of the inorganic surrogate. It is most certainly worth publishing for this reason alone.

Since hypoxic conditions in tumours are taken to be predictive of a poor clinical outcome, the authors tested whether the direction of changes in the transcriptome of their untreated and hypoxic cell lines mirrors the difference between tumours and their normal cell counterparts. Hypoxia scores from mRNA expression data were somewhat (but significantly) elevated in recurrent/progressive patients.However, this difference does not (unfortunately) appear to be sufficient to use clinically (if I am understanding their intentions correctly). The upshot is that the authors do not mention this comparison in their Discussion.

On practical matters: they might expand the caption to Fig 1. In A) I assume the three tracks are biological replicates: if so, write it. In B) a fuller explanation of the X-axis ‘Index’ would be helpful. Check on the printing of ‘300mM CoCl _2_’ (in Methods, bottom of p2).

I have read this submission. I believe that I have an appropriate level of expertise to confirm that it is of an acceptable scientific standard.

# Peer review: Referee response for version 1

**DOI:** 10.5256/f1000research.8152.r11723

**Published:** 2016-01-04

**Authors:** Alexey Ruzov

**Affiliations:** 1 Division of Cancer and Stem Cells, School of Medicine, Centre for Biomolecular Sciences, University of Nottingham, Nottingham, UK

In this paper, the authors analysed the response of renal cancer cells Caki-1 to hypoxia and CoCl _2 _treatment using whole transcriptome sequencing. The main finding of the manuscript that the profiles of gene expression of Caki-1 cells are qualitatively different under these two conditions, and, therefore, the CoCl _2 _treatment may not represent an adequate model mimicking hypoxia, is interesting and merits indexation, in my opinion.

Although, as far as I can ascertain, most of the experiments are technically sound and the main conclusions of the study are justified, I feel that there is a number of issues with the text/interpretation of the data, which need to be addressed to improve the quality of the manuscript:

1. It is not sufficiently clear from the text why the authors have elected the Caki-1 cell line for their study. Is there anything peculiar about these cells? Is VHL mutated/lost/misexpressed in these cells? The last question is especially important since the authors mention that this protein  is lost in 50-70% of renal cell carcinomas.
2. According to the Dataset 1, the authors used triplicates for each condition in their analysis. This should be stated in Methods section. The authors should also comment on the nature (biological vs technical) of these replicates there.
3. It is not clear if the numbers presented in the Venn diagram in Figure 2A represent all genes up/down regulated for each condition or only the genes enriched for the indicated KEGG pathways. It is also not clear from the annotation or legend which numbers correspond to upregulated and downregulated genes in this figure.
4. The Discussion is very weak at the moment with no references cited in the section. The authors should rewrite it relating their findings to available literature. 

Other points:

1. The abstract lacks a sentence summarizing results of the study and/or pointing at potential implications of the findings for medicine/cancer biology. It would be beneficial for readers if such statement was included in the abstract.
2. The last sentence in Introduction (starting with "We propose...") may well be an overinterpretation. The conclusion that CoCl _2 ^^_treatment is not a "correct model for hypoxia" may need to be additionally confirmed using other cell line systems.
3. Presenting the KEGG enrichment scores for each category alongside the Venn diagram may be helpful for assessing significance of the results. 

I have read this submission. I believe that I have an appropriate level of expertise to confirm that it is of an acceptable scientific standard.
